# Enhancing the Bothropic Antivenom through a Reverse Antivenomics Approach

**DOI:** 10.1021/acs.jproteome.4c01028

**Published:** 2025-01-22

**Authors:** Tassia Chiarelli, Jackelinne Y. Hayashi, Nathalia da Costa Galizio, Fernanda M. S. Casimiro, Ricardo Torquato, Aparecida S. Tanaka, Karen de Morais-Zani, Anita M. Tanaka-Azevedo, Alexandre K. Tashima

**Affiliations:** † Departamento de Bioquímica, Escola Paulista de Medicina, Universidade Federal de São Paulo, São Paulo 04023-901, Brazil; ‡ Laboratório de Fisiopatologia, Instituto Butantan, São Paulo 05503-900, Brazil; § Laboratório de Herpetologia, Instituto Butantan, São Paulo 05503-900, Brazil

**Keywords:** antivenom, Bothrops jararaca, snakebite, reverse antivenomics, affinity chromatography, proteomics

## Abstract

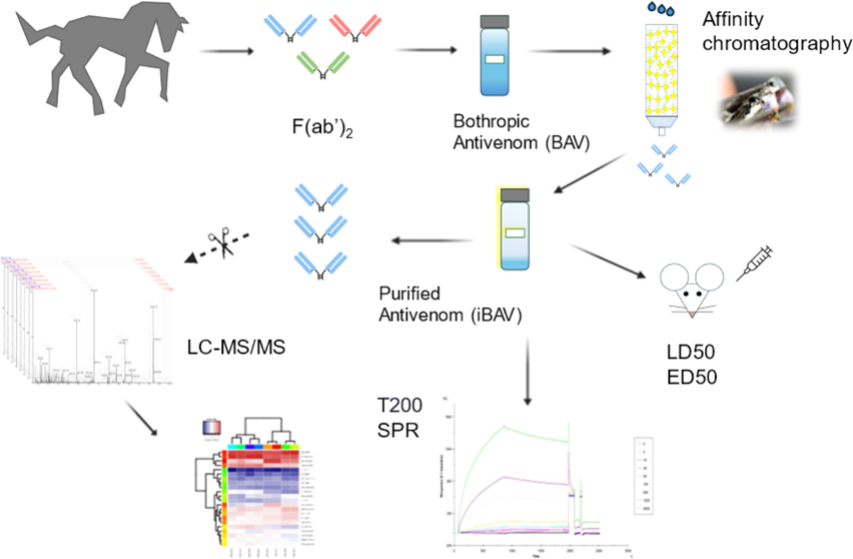

Antivenoms are the only effective treatment for snakebite envenomation and have saved countless lives over more than a century. Despite their value, antivenoms present risks of adverse reactions. Current formulations contain a fraction of nonspecific antibodies and serum proteins. While new promising candidates emerge as the next generation of antivenoms, it remains clear that animal-derived antivenoms will still play a critical role for years to come. In this study, we improved the bothropic antivenom (BAv), by capturing toxin-specific antibodies through affinity chromatography using immobilized *Bothrops jararaca* venom toxins. This process produced an improved antivenom (iBAv) enriched in neutralizing antibodies and depleted of serum proteins. Proteomic analysis showed that iBAv was 87% depleted in albumin and 37–83% lower in other serum proteins compared to BAv. Functional evaluation demonstrated that iBAv had a 2.9-fold higher affinity for venom toxins by surface plasmon resonance and a 2.8-fold lower ED50 *in vivo*, indicating enhanced potency. Our findings indicate that enriching specific antibodies while depleting serum proteins reduces the total protein dose required and increases the potency of antivenom. Although technical and economic considerations remain for large-scale implementation, this affinity-enriched antivenom represents a significant advancement in improving antivenom efficacy against *B. jararaca* envenomations.

## Introduction

Worldwide estimates of snakebite envenoming reach more than 5 million cases every year, resulting in more than 100,000 deaths and around 400,000 people with permanent disabilities or disfigurements such as amputation, necrosis, and severe local damage.^1−3^ In addition to the public health problem, snakebite envenomation carries a significant economic burden, as most victims are young agricultural workers and children from poor communities.^1,4^ The lack of sufficient antivenom supplies in regions such as sub-Saharan Africa and parts of Asia has exacerbated this problem, prompting the World Health Organization (WHO) to add snakebite envenoming to the list of Neglected Tropical Diseases in 2017.^5,6^

Since the late 19th century, antivenoms produced from the serum of immunized animals have been the only effective and scientifically validated treatment for snakebite envenoming.^7,8^ Antivenom production involves administering sublethal doses of snake venom to animals, which in turn produce the necessary antibodies to neutralize the venom toxins.^9^ Antibodies from animal serum then form the core of the antivenom therapy. Despite their lifesaving value, antivenoms are not free of adverse reactions. Hyperimmune animal plasma contains not only neutralizing antibodies but also other antibodies that are part of the animal’s immune repertoire, along with other plasma proteins.^10,11^ While manufacturers increase the purity of antivenoms using processes to enrich immunoglobulins and remove plasma proteins, nonspecific antibodies still remain and can contribute to the onset of adverse reactions in treated patients.^11^ It is estimated that only 10–40% of antibodies in most antivenoms neutralize venom toxins.^11−13^

In response to these challenges, new technologies and alternative therapeutic approaches for antivenoms are under extensive investigation. Promising candidates, such as monoclonal antibodies against -neurotoxins,^14,15^ nanobodies,^16^ small molecules,^17,18^ and *de novo* designed protein inhibitors^19^ have shown remarkable results. While these approaches have shown significant promise, they may take considerable time to undergo clinical trials, gain regulatory approval, and scaling up for industrial production.^20^ Therefore, improving current antivenom therapies and ensuring their accessibility remain critical priority.

Previous studies have demonstrated that using affinity chromatography with venom toxins to enrich specific immunoglobulins increases the potency of purified antivenom.^21−24^ This process has been scaled up by manufacturers such as SERB Pharmaceuticals (CroFab, against North American pit viper envenomation, https://crofab.com/About-CroFab/Manufacturing), and MicroPharm (ViperaTab, against *Vipera berus* envenomation^25^). However, to the best of our knowledge, no study has yet provided a quantitative proteomic characterization of crude and affinity-purified antivenoms or examined the extent of serum protein depletion and antibody enrichment achieved through this methodology. Furthermore, the potential improvement of the bothropic antivenom using this approach has not yet been demonstrated.

Advances in the characterization of immune reactivity of antivenoms have been achieved using the proteomic-based protocol known as antivenomics.^13,26^ In its third generation, this method involves immobilizing antivenom components on an affinity column and quantifying its cross-reactivity by analyzing bound and unbound venom toxins.^13^ In this study, we used a reverse antivenomics approach in which venom toxins were immobilized to selectively bind and enrich specific antibodies. Using this method, we enriched bothropic antivenom (BAv) antibodies against *Bothrops jararaca* venom to produce an improved bothropic antivenom (iBAv). The BAv and iBAv were subsequently characterized by proteomics and *in vitro* and *in vivo* assays.

## Materials and Methods

### Ethical Aspects

This study has been approved by the Ethics Committee on Animal Use of the Federal University of São Paulo (CEUA/UNIFESP) under approval number 5190180218. Additionally, it has been approved by the Ethics Committee on Animal Use of Instituto Butantan (CEUAIB), where part of the project was conducted, under approval number 6577260218. Access to genetic heritage was registered in the SisGen system, in compliance with Brazilian law 13123/2015 under the registration number A794BDF.

### Venom of *B. jararaca* and Bothropic Antivenom

Brazilian Bothropic Reference Venom (BBRV) was produced and donated by the Instituto Butantan (São Paulo, Brazil). It was the fifth batch (BRA/BOT/05), composed of pooled venoms from the first extractions from 4,430 *B. jararaca* snakes donated to Instituto Butantan. The snakes were from the Brazilian states of São Paulo (49.2%), Espírito Santo (24.2%), Santa Catarina (15.6%), Paraná (10.1%), Minas Gerais (0.8%), and Rio de Janeiro (0.1%). Most of the specimens were adults. Venom extraction was carried out at the Herpetology Laboratory, Instituto Butantan. The animals were anesthetized with carbon dioxide, and venom was collected by manual compression of their glands. The venoms were centrifuged at 16,000*g* for 5 min at 4 °C, lyophilized, and stored at −20 °C. Pentavalent bothropic antivenoms were obtained from Instituto Butantan (BAv, batch 1: number 16006, expiration date 03/2019; batch 2:1305077, expiration date 05/2013). The production process involves immunizing horses with antigens prepared by a mixture of venom pools from *B. jararaca* (50%), *Bothrops alternatus* (12.5%), *Bothrops jararacussu* (12.5%), *Bothrops moojeni* (12.5%), and *Bothrops neuwiedi* complex (12.5%). Once the immunized animals develop appropriate immune responses, blood is partially withdrawn, enzymatically processed with pepsin, and purified to obtain *F*(ab’)_2_ fragments. The nominal neutralizing potency is 5 mg of neutralized *B. jararaca* reference venom per milliliter of antivenom, according to the official antivenom label.

### Animals

For *in vivo* experiments, Swiss male mice weighing between 18 and 22 g were used, provided by the central animal facility of Instituto Butantan. The animals had *ad libitum* access to water and food and were kept on a 12 h light/dark cycle. Surviving animals were euthanized in a CO_2_ chamber. Experiments were conducted using 5 animals per group.

### Determination of Protein Concentration

The concentration of proteins in solution was determined using Bradford reagent (Sigma-Aldrich, B6916) with bovine serum albumin as the standard (Thermo Scientific 23225), according to the manufacturer’s protocol. Absorbance readings were performed at 595 nm using a Synergy HT spectrophotometer (BioTek).

### Purification of Bothropic Antivenom by Affinity Chromatography

The affinity column was prepared using CNBr-activated Sepharose 4B resin (GE Life Sciences). An aliquot of 210 mg of the resin was suspended in 1 mM HCl and 2.5 mg of *B. jararaca* venom was dissolved in 0.4 M NaHCO_3_ binding buffer (pH 8.3) containing 1 M NaCl. The binding solution, containing the dissolved venom, was added to the resin and allowed to react overnight at 4 °C to covalently bind the free amine groups of the toxins to the resin. After the reaction, excess venom was removed, and the resin was washed 3 times alternating 0.1 M Tris-HCl (pH 8.5) and 0.1 M acetate (pH 4.0) containing NaCl 0.5 M, to block the remaining active groups and remove uncoupled ligands.

Preliminary loads were performed on the affinity column using amounts ranging from 0.2 to 2.4 mg of BAv to verify the column saturation. After a linear recovery trend of BAv was verified throughout the range, all subsequent purifications were carried out using 2.4 mg of BAv. The antivenom was loaded onto the venom toxin column using 50 mM Tris-HCl, 1 mM CaCl_2_ (pH 8) as the binding buffer. Following BAv application, the column was washed with 50 mM Tris-HCl, 1 mM CaCl_2_ (pH 8) to remove the unbound compounds (flow-through). The bound immunoglobulins were eluted with 100 mM glycine-HCl and 0.5 M NaCl (pH 2.7), and this eluate composed the iBAv. The pH of the collected eluate was then adjusted to 7.0 with 0.1 M Tris-HCl (pH 9.0). The column was re-equilibrated with 50 mM Tris-HCl and 1 mM CaCl_2_ (pH 8) for subsequent purifications. After elution, the buffers were exchanged for 0.9% NaCl saline solution using Amicon 10 kDa centrifuge filters (Millipore). The purified antibodies were stored at 4 °C until subsequent analyses.

Recovery yields of the retained eluates were calculated using the following formula:
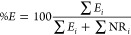
where, *%E* is the percentage recovery yield of F(ab’)_2_ retained in the affinity column, *E*_*i*_ is the amount of protein (mg) in each retained eluate fraction *i*, and NR_*i*_ is the amount of protein (mg) of the nonretained fraction *i*, which includes the loading solution, flow-through, and washing solutions.

### In Solution Trypsin Digestion

The digestions of BAv and iBAv (batch 1 and 2) were carried out using 100 μg of each sample diluted in 50 mM NH_4_HCO_3_. Disulfide bonds were reduced with 5 mM dithiothreitol for 30 min at 60 °C and subsequently alkylated with 15 mM iodoacetamide for 30 min at room temperature in the dark. Proteins were digested overnight at 37 °C using trypsin (V5511, Promega) at a 1:100 ratio (enzyme/substrate). The samples were then cleaned up using reversed-phase C18 StageTip columns.^27^ The eluates were dried by using a vacuum centrifuge and stored at −20 °C until analysis.

### Mass Spectrometry

Aliquots of digested peptides from each sample were analyzed using an UltiMate 3000 RSLCnano liquid chromatography system (Thermo Fisher Scientific) coupled to an Impact II mass spectrometer (Bruker Daltonics), equipped with a CaptiveSpray ionization source. The samples were injected into a 75 μm × 15 cm Acclaim PepMap C18 column (Thermo Fisher Scientific). Chromatographic separation was performed using a gradient of 5 to 40% phase B (0.1% formic acid in 80% ACN) with a flow rate of 250 nL/min for 90 min. Liquid chromatography mass spectrometry (LC-MS/MS) data were acquired using data-dependent acquisition, selecting the 8 most abundant precursor ions per cycle with precursor intensity-adjusted MS/MS acquisition time. Precursor ions were acquired in the *m*/*z* range of 300–1750, with a dynamic exclusion time set at 0.4 min. Collision energies were adjusted between 23 and 65 eV. Each sample was analyzed in duplicate.

### Database Search and Quantification

Mass spectrometry data were loaded, processed, and aligned using Progenesis QI for Proteomics (Nonlinear Dynamics) as previously described.^28^ Briefly, the LC-MS/MS files for each analysis were imported, and a reference run was automatically selected for all runs. Precursor ions were submitted to retention time alignment and isotopic peak picking and normalized to the reference run using default parameters. Peptide ion abundances were calculated as the sum of areas under the isotope boundaries and then normalized by a gain factor to correct for systematic experimental variations. After processing, .mgf files containing MS/MS spectra (limited to 1000 fragments per spectrum) were exported to PEAKS Studio X+ (Bioinformatics Solutions Inc.) for database searching against a custom protein database comprising 294 *B. jararaca* sequences and 20,869 horse (*Equus caballus*) sequences obtained from UniProtKB (www.uniprot.org, downloaded on Dec 29, 2022). Searches were performed using carbamidomethylation of Cys as a fixed modification, oxidation of Met as a variable modification, and semispecific trypsin digestion was set, allowing up to two missed cleavages. Mass tolerances were configured at 50 ppm for precursor ions and 0.05 Da for fragment ions, with a maximum of two variable modifications permitted per peptide. The false discovery rate (FDR) was set at 1%, estimated via the decoy fusion method.^29^ The resulting pep.xml identification files were exported back to Progenesis QI for Proteomics for quantification. Relative quantification was performed using the average signal response of unique nonconflicting tryptic peptides per protein, considering a minimum of 2 unique peptides. Protein abundances in each run were normalized to percentages of total intensities of each run. Fold changes were calculated as ratios of protein levels in iBAv/BAv. Statistical significance was determined via ANOVA, with *p*-values <0.05 considered significant. Pie charts were created in OriginPro 2016 (OriginLab), while heatmaps were generated in R (https://www.r-project.org/) using the gplots, RColorBrewer, and corrplot packages.

### Surface Plasmon Resonance

Molecular interactions among *B. jararaca* venom toxins and antivenom antibodies were assessed by using surface plasmon resonance (SPR) on a Biacore T200 (GE Healthcare). Approximately 200 μg of venom toxins were dissolved in 10 mM sodium acetate buffer (pH 5.5) and immobilized on a CM5 Sensor Chip (GE Healthcare), by amine coupling using HBS-N buffer (GE Life Sciences), following the manufacturer’s instructions. Unbound toxins were removed by injecting 10 mM glycine at pH 2.0. The immobilized toxins were subsequently incubated with BAv and iBAv (batch 1) at varying protein concentrations ranging from 5 to 4000 nM (estimated based on the molecular mass of F(ab’)_2_, approximately 90 kDa) on the chip surface. The concentrations used were 5, 10, 30, 50, 100, 300, 600, 1200, and 4000 nM. BAv and iBAv were injected at a flow rate of 10 μL/min for 800 s in the association phase, followed by a dissociation phase of 1000 s. Surface regeneration was performed by injecting 2.0 M glycine-HCl (pH 2.0) for 100 s at a flow rate of 30 μL/min, followed by a wash with 2.0 M NaCl at the same flow rate.

The results were analyzed using Biacore T200 Evaluation Software (v1.0). Sensorgrams were processed by subtracting the reference value from the chip containing immobilized toxins without any interaction with any molecules. Binding kinetics parameters were measured according to the 1:1 Langmuir model.^30^ The equilibrium dissociation constants (*K*_D_) were calculated as the quotient of the dissociation constant (*k*_d_) over the association rate (*k*_a_) and were used to compare the interaction profiles of BAv and iBAv.

### Median Effective Dose (ED50)

The median effective dose (ED50) was used to estimate the neutralizing efficacy of the antivenoms. It is defined as the dose of antivenom capable of neutralizing the action of venom in 50% of a specific population of mice based on the amount of venom that induces the 50% lethal dose (LD50). The ED50 of the original bothropic antivenom (BAv) and of the improved bothropic antivenom (iBAv) was determined by incubating serial dilutions of the antivenoms at 37 °C for 30 min with a fixed dose corresponding to 5 LD50 (180 μg) of Brazilian Bothropic Reference Venom (BBRV, BRA/BOT/Lot 05, Instituto Butantan). The total volumes were adjusted to 500 μL with 0.9% NaCl saline solution.

To normalize the conditions, the volume of each antivenom required was calculated based on protein concentration, and the range of dilutions was varied to achieve a suitable spectrum of neutralizing doses for condition. The venom/antivenom mixture was then injected intraperitoneally (i.p.) into mice groups, with 5 mice per antivenom dilution.^31,32^ Control groups were inoculated i.p. only with saline as well as with the highest doses of BAv and iBAv mixed with sterile saline. The effective dose was calculated based on the number of deaths within 48 h after injection of the venom/antivenom mixture using Probit analysis, expressed as μg antivenom per animal.^33^

## Results

### Affinity Chromatography

**Figure 1:**
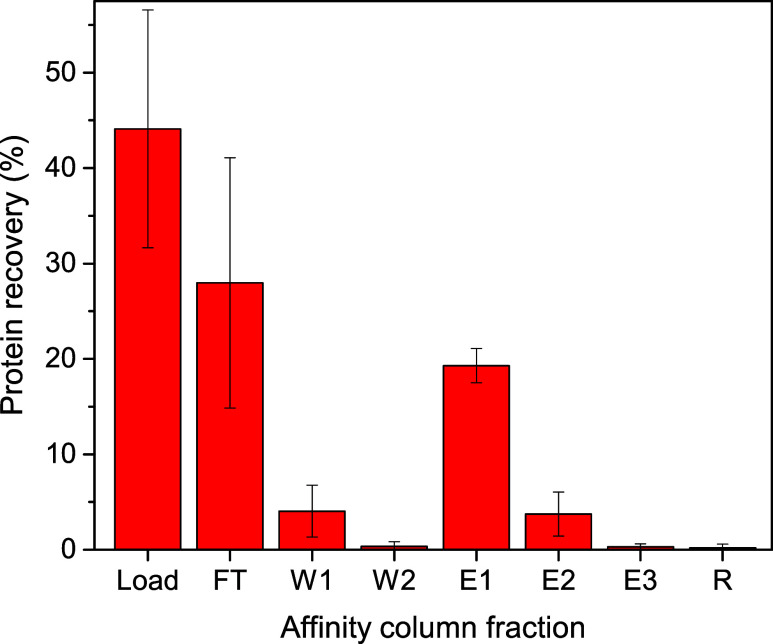
Affinity chromatography eluates of bothropic antivenom (BAv) on a *B. jararaca* venom toxin CNBr-activated column. Fractions were collected after loading BAv in 50 mM Tris-HCl solution, 1 mM CaCl_2_, pH 8.0, followed by washing the column with 50 mM Tris-HCl, 1 mM CaCl_2_, pH 8.0. The load eluate (Load), flow-through (FT), and consecutive washes (W1–W2) contained unbound proteins. Proteins bound to the column were eluted with 100 mM glycine-HCl, 0.5 M NaCl, pH 2.7 (E1–E3), forming the improved bothropic antivenom (iBAv) fractions. The column was re-equilibrated with 50 mM Tris-HCl buffer and 1 mM CaCl_2_, pH 8.0 (R) for subsequent chromatography.

Bothropic antivenom batches produced by Instituto Butantan were subjected to affinity chromatography to purify specific antibodies against *B. jararaca* venom toxins linked to a CNBr-activated Sepharose column. Protein quantification of the chromatographic fractions revealed that, on average, 72.2% (95% CI: 64.7–79.8%) of BAv components did not interact with the immobilized toxins and were collected in the flow-through fractions and subsequent washes (Figure 1). The components that interacted and were noncovalently bound to the toxins were eluted with glycine-HCl buffer (pH 2.7), corresponding to 27.8% (95% CI: 20.2–35.3%) of the total BAv proteins (Figure 1). These bound proteins composed the improved bothropic antivenom (iBAv).

### Quantitative Proteomic Analysis

**Figure 2:**
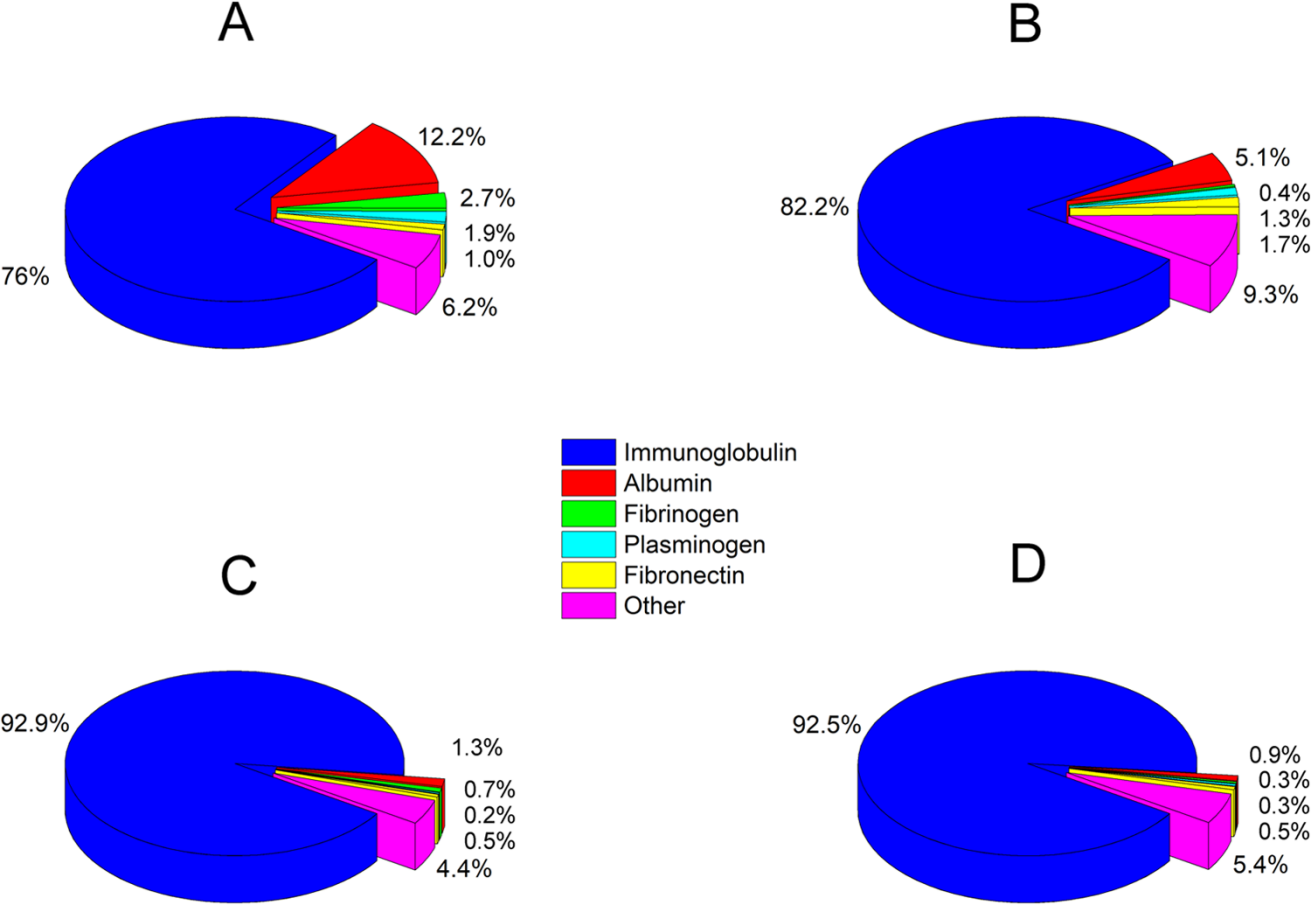
Composition of the original (BAv) and improved bothropic antivenom (iBAv) proteins as determined by quantitative proteomic analysis: (A) BAv–batch 1; (B) BAv–batch 2; (C) iBAv–batch 1; (D) iBAv–batch 2.2.

BAv and iBAv samples (batches 1 and 2) were digested with trypsin and submitted to data-dependent acquisition LC-MS/MS analysis for proteomic identification and label free quantification of antivenom proteins. The database search of MS/MS spectra resulted in the identification of 3270 peptides from 102 proteins. A total of 49 proteins were quantified through label free quantification (Table S1). The results indicated that BAv is composed, on average, by 79.1% of immunoglobulins, including IgG, IgA (0.9%), and IgM (0.9%) heavy chains and kappa and lambda light chains (Table 1 and Figure 2). Horse albumin was the second most abundant protein, accounting for 8.6% of BAv. Other relevant serum proteins included fibrinogen, plasminogen, and fibronectin, representing 1.5, 1.6, and 1.3% of BAv, respectively (Table 1 and Figure 2). Collectively, all other proteins accounted for an average of 7.8% of BAv. Both batches of BAv exhibited high contents of immunoglobulins, while the composition of other proteins identified in horse serum varied (Table 1).

**Table 1:** Average Abundances of Protein Classes Observed by Quantitative Proteomics Analysis of BAv and iBAv

protein class	Bav (%)	iBAv (%)	fold change	depletion/enrichment (%)
immunoglobulin	79.1	92.7	1.17	17.1
albumin	8.6	1.1	0.13	–87.0
fibrinogen	1.5	0.5	0.34	–65.9
plasminogen	1.6	0.3	0.17	–83.3
fibronectin	1.3	0.5	0.37	–62.5
other	7.8	4.9	0.63	–36.6

**Figure 3:**
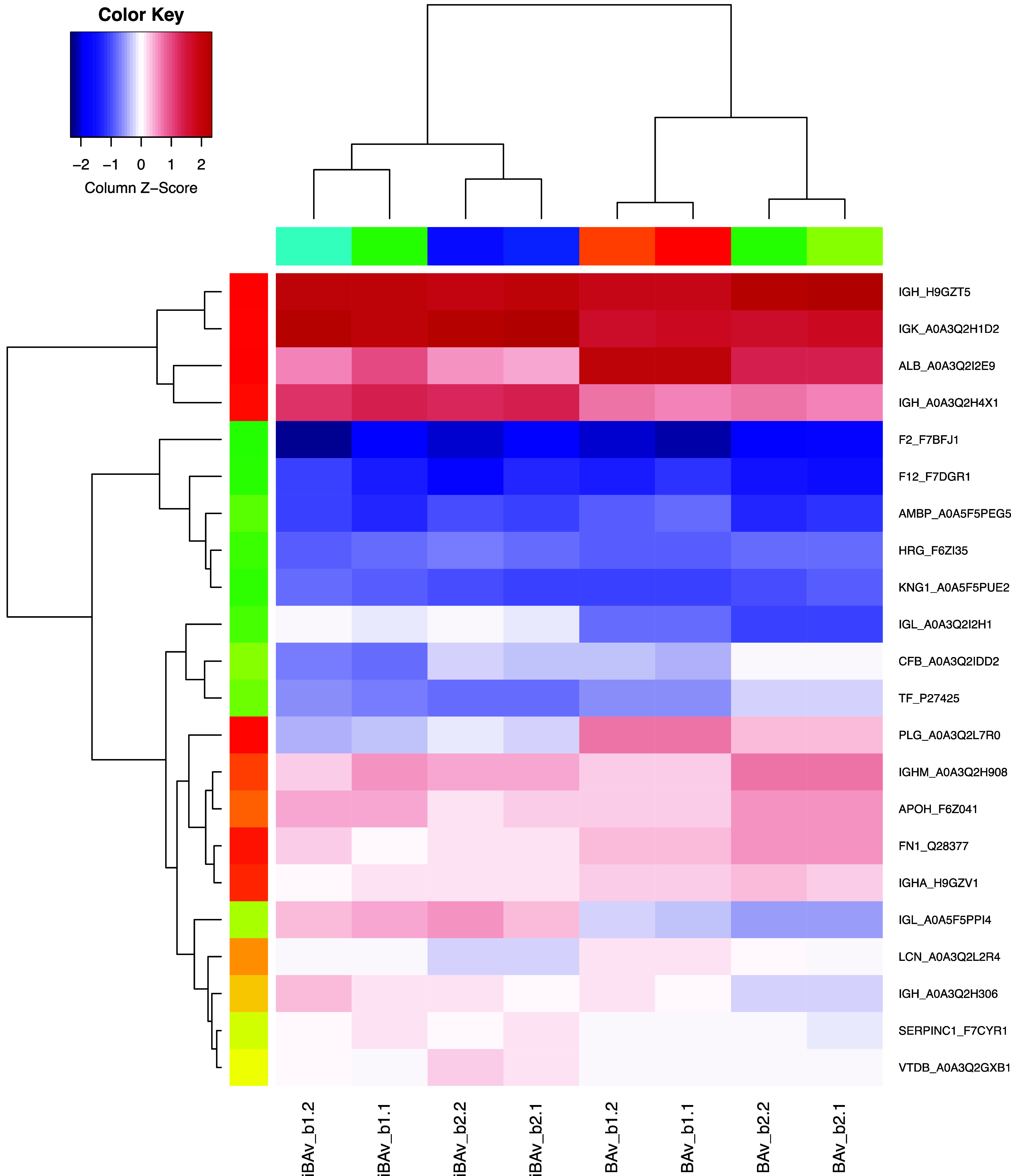
Heatmap illustrating the significantly changed proteins between BAv and iBAv (*p* < 0.05). Batches are indicated by the first number and LC-MS/MS replicates by the second number (*e.g.*, BAv_b1.2 refers to batch 1 and replicate run 2).

After submitting the BAv batches to affinity chromatography, nonspecific immunoglobulins and serum proteins were depleted, resulting in a more homogeneous composition in the iBAv batches (Figure 2 and Table S1). The average composition of immunoglobulins in iBAv increased to 92.7%, while the fraction of horse albumin decreased to 1.1% (Table 1). The levels of fibrinogen, plasminogen, fibronectin, and other remaining proteins were reduced to 0.5, 0.3, 0.5, and 4.9%, respectively (Table 1 and Figure 2). The fold changes of 25 out of the 49 quantified proteins were statistically significant (Figure 3 and Table S1). Notably, all serum protein levels decreased in the iBAv batches, while most immunoglobulins increased (Figure 3 and Table S1). The most significant reductions in the iBAv in comparison to BAv were observed for albumin (∼8-fold), plasminogen (∼6-fold), serotransferrin (∼3-fold), fibrinogen (∼3-fold), fibronectin (∼3-fold), prothrombin (∼3-fold), and complement factor B (∼3-fold) (Tables 1 and S1).

### Interaction Kinetics by Surface Plasmon Resonance

**Figure 4:**
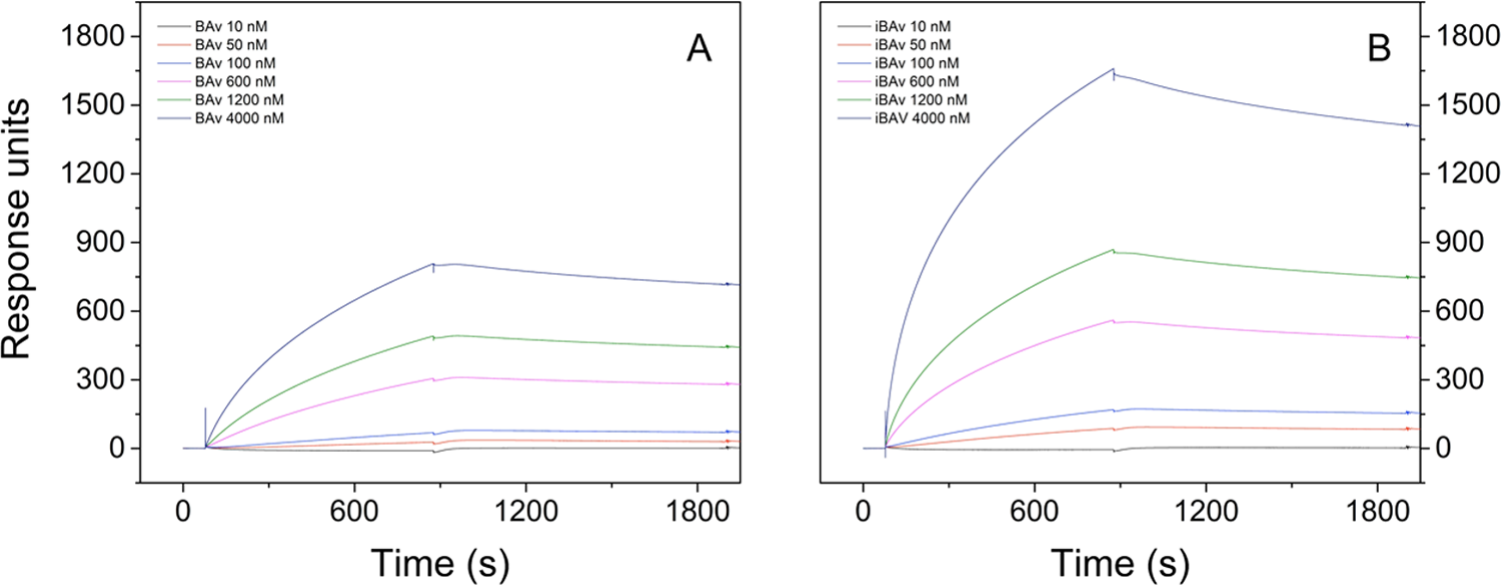
Sensorgram of surface plasmon resonance assays showing the association and dissociation profiles of antivenom antibodies binding to *B. jararaca* venom toxins. Response units were measured at concentrations ranging from 10 to 4000 nM. (A) BAv; (B) iBAv.

The kinetic parameters for the interaction between BAv and iBAv with toxins from *B. jararaca* venom were determined by using surface plasmon resonance (SPR) on a Biacore T200. Samples were injected at concentrations ranging from 5 to 4000 nM. The interaction kinetics are presented in Figure 4. The association rate constant (*k*_a_), dissociation rate constant (*k*_d_), and equilibrium dissociation constant (*K*_D_) for BAv and iBAv at 25 °C are presented in Table 2. The SPR data indicate that both antivenoms exhibit high-affinity interactions with *B. jararaca* venom, as indicated by their dissociation constants (*K*_D_) in the nanomolar range (10^–7^–10^–9^), consistent with high-affinity binding.^34^ Notably, iBAv exhibited a *K*_D_ value that was 2.9 times lower than that of BAv. This result was driven primarily by a significantly higher association rate (*k*_a_) of iBAv, which indicates a stronger and more efficient binding to the venom components (Table 2). The dissociation rates (*k*_d_) for both antivenoms were similar, implying comparable stability of the antibody-toxin complexes once formed, indicating that iBAv binds to the venom with a significantly higher affinity.

**Table 2:** Kinetic and Equilibrium Constants for Association and Dissociation of BAv and iBAv Components to Bothropic Venom Toxins Immobilized in an SPR Chip at 25 °C

kinetic constants (25 °C)	BAv	iBAv
*k*_a_ (M^–1^ s^–1^)	562.2	1877.0
*k*_d_ (s^–1^)	1.056 × 10^–4^	1.210 × 10^–4^
*K*_D_ (M)	1.879 × 10^–7^	6.447 × 10^–8^

### Effective Dose 50% (ED50)

The potency of iBAv compared to BAv in neutralizing the lethal activity of *B. jararaca* venom was assessed in mice. The ED50, representing the dose of antivenom required to protect 50% of the animals, was expressed in μg of antivenom per animal. The ED50 for BAv was 139.9 μg/animal (95% confidence interval: 102.1–179.2 μg/animal), while for iBAv, it was significantly lower at 49.5 μg/animal (95% confidence interval: 28.4–79.5 μg/animal). Although both antivenoms effectively neutralized the venom’s lethal toxicity, iBAv demonstrated a 2.8-fold higher potency compared to BAv. The percentage of deaths in each group, based on the amount of antivenom protein administered, is presented in Table 3. No deaths were observed in the control groups injected with only saline, BAv and iBAv.

**Table 3:** *In Vivo* Neutralization Capacity of BAv and iBAv Against 5 LD50 (180 μg) of Brazilian Bothropic Reference Venom (BBRV) in Mice to Determine ED_50_ by Probit Analysis

antivenom	groups^a^	antivenom (μg/mouse)	deaths (%)^b^	ED50 (μg/mouse)^c^	potency ratio
BAv	1a	342.0	0	139.9 (102.1–179.2)	1
	2a	244.3	0		
	3a	174.5	0		
	4a	124.6	100		
	5a	89.0	80		
iBAv	1b	256.5	0	49.5 (28.4–79.5)	2.83
	2b	171.0	0		
	3b	85.5	0		
	4b	34.2	100		
	5b	17.1	100		

a Groups 1a–5a received BAv, and 1b–5b received iBAv.

b percentage of deaths in groups of mice receiving increasing dilutions of BAv or iBAv antivenoms mixed with 5 LD50 of BBRV via i.p. route (*n* = 5) within 48 h after injection of the mixture.

c ED50 with the 95% confidence interval.

## Discussion

The bothropic antivenoms produced by Instituto Butantan, Instituto Vital Brasil, and FUNED remain the only valid therapies for treating envenomation caused by snakes of the *Bothrops* genus in Brazil. However, previous studies demonstrated that only a fraction of antibodies in antivenoms specifically target venom toxins.^11,13,35^ By using affinity chromatography with venom toxins immobilized on CNBr-activated Sepharose resin, we were able to capture specific antibodies while removing nonspecific antibodies and serum proteins. As a result, through this reverse antivenomics approach, we produced and characterized an improved bothropic antivenom enriched with neutralizing antibodies, which demonstrated enhanced potency and efficacy in comparison to those of the original bothropic antivenom.

Approximately 28% of the F(ab’)_2_ in the bothropic antivenom is specific to *B. jararaca* venom toxins, a proportion similar to that observed in other antivenoms characterized through the antivenomics approach.^12,13,35^ Sanz et al. reported 24% of *B. jararaca* venom toxin-binding F(ab’)_2_ in BAv,^35^ aligning with the 95% CI (20–35%) of the present study. It is notable that despite using a different methodology, we obtained similar yields. In third generation antivenomics affinity chromatography, antivenom IgG or F(ab’)_2_ molecules are immobilized on a CNBr-activated resin, to which snake venom toxins bind and are subsequently eluted.^13^ Toxin quantification is then performed by integrating RP-HPLC peak areas based on absorbance at 215 nm. In contrast, our reverse antivenomics involves immobilizing venom toxins on the CNBr-activated resin, allowing the antivenom F(ab’)_2_ to bind to these toxins before elution. The eluates were then quantified using the method of Bradford.

Pla et al. reported that 15% of antibody molecules in EchiTAb-Plus-ICP antivenom, developed against the African snake *Bitis arietans*, specifically target venom toxins.^13^ Similarly, in antivenoms formulated against *Bothrops asper*, *Crotalus simus*, and *Lachesis stenophrys* venoms, 10–40% of antibodies were found to target venom toxins.^11^ The proportion of active components of BAv falls within the range observed in these previous antivenom studies. It has been suggested that antivenoms effectively neutralize venoms when they capture approximately 20–25% of venom components.^36^ This indicates that a significant portion of immunoglobulins and other impurities in antivenoms, including BAv, are not venom-specific and do not contribute to toxin neutralization.

As expected, proteomic analysis showed that the primary components of BAv were horse immunoglobulins, comprising 79.1% of the total protein content, although significant amounts of horse serum proteins were also detected. The immunoglobulin content aligns closely with that of the trivalent antivenom SABU from Indonesia, which consists of 84.0% immunoglobulins.^37^ The main contaminant of SABU was also serum albumin, accounting for 5.5% of antivenom proteins, while in BAv, it accounted for 8.6% (Tables 3 and S1). The composition of BAv was also similar to polyvalent antivenoms (PAV) produced in India, in which the proportion of immunoglobulins varied between 78.7 and 94.9%.^38,39^ Tan et al. employed a methodology based on peak intensities from size exclusion chromatography combined with mass spectrometry identification of proteins in fractions,^37^ whereas Patra et al. used proteomics identification followed by label free quantification,^38^ similar to the approach used in this study. It is important to note that the database used in this work contains sequences of horse immunoglobulins which do not completely align with the actual F(ab’)_2_ antibodies, as the complementarity-determining regions are highly variable and may account for 15–20% of the variable domains.^40^ The lack of a comprehensive horse antivenom IgG database may have limited the identifications and quantification of F(ab’)_2_, although our results showed the expected trends, with immunoglobulin enrichment and serum protein depletion in iBAv.

Serum proteins are the components of animal-derived antivenoms most likely to elicit immune responses.^41^ Animal antisera are highly associated with early adverse reactions (EAR) and late serum reactions.^42^ Although advances in production and purification processes have significantly reduced the risk of EAR, it has still been reported to occur between 5 and 57% of cases, with life-threatening anaphylaxis in approximately 1% of cases.^4^ In addition to albumin, other proteins were identified in BAv, including fibrinogen (1.5%), plasminogen (1.6%), fibronectin (1.3%), and other serum proteins (7.8%) (Table S1). These serum components do not neutralize venom toxins and were present in BAv at levels similar to those reported in other antivenom studies.^11,13^ The enrichment of specific F(ab’)_2_ by affinity chromatography reduced the albumin content in iBAv by 87%, and other serum components by 37–83% (Table 1), according to the proteomics data (Table S1).

*In vitro* surface plasmon resonance and *in vivo* neutralization assays of BAv and iBAv produced convergent results. SPR demonstrated that iBAv F(ab’)_2_ had 2.9 times higher affinity for *B. jararaca* venom toxins than BAv, while *in vivo* neutralization assays showed that the ED50 of iBAv was 2.8 times lower than that of BAv, indicating significantly higher effectiveness in neutralizing the lethal activity of *B. jararaca* venom in mice. These results closely align with the improvement of ED50 of affinity-purified ovine antivenom against *V. berus*, reported by Smith et al.^23^ SPR provided valuable information about the kinetics of antibody–antigen binding events. In fact, neutralizing antibodies in the original BAv were enriched in iBAv, and as a result, their relative concentration increased and produced a stronger association with venom toxins supported in the SPR sensor chip. The *in vivo* neutralization assay further demonstrated that iBAv, depleted of serum proteins and nonspecific F(ab’)_2_, was more effective in neutralizing the lethal activity of *B. jararaca* venom. That is, iBAv achieved a neutralization effect similar to that of BAv with approximately one-third of the protein dose.

The neutralizing activity of antivenoms is currently evaluated through *in vivo* observations in animal models, and supportive evidence for clinical efficacy is largely extrapolated from these studies.^43,44^ Clinically, increasing the concentration of specific neutralizing antibodies may allow for lower doses of heterologous proteins in patients, potentially reducing the risk of adverse side effects.^45^ As previously mentioned, animal-derived antivenoms are frequently associated with adverse reactions due to cross-species reactivity.^4,46−48^ While iBAv may represent a safer alternative, our study was not designed to assess the side effects and safety of this treatment, which remains an important area for future investigation.

Another important point regarding our methodology is that the family of bradykinin potentiating peptides (BPPs) is under-represented in the pool of affinity-purified F(ab’)_2_. BPPs have a pyroglutamic acid at their N-terminus, and most of them lack lysines in their sequences. As a result, these peptides do not have free amines available to couple to the CNBr-activated resin. Consequently, antibodies against BPPs are not expected to be found using our approach. Additionally, *in vivo* neutralization assays, involving preincubation of venom and antivenom in mice, do not perfectly replicate natural envenomation, as venom and antivenom are mixed before injection. However, this approach remains the gold standard for assessing antivenom potency.

We have demonstrated the feasibility of producing an improved bothropic antivenom, enriched in specific F(ab’)_2_ and depleted in serum proteins, as demonstrated through proteomic analysis. The iBAv displayed a higher potency compared to the original BAv. However, implementing it as a therapeutic product would require a technical and economic evaluation, which is beyond the scope of this study. On the one hand, adding a purification step to the production process would increase manufacturing costs and require a new validation process for regulatory agencies. On the other hand, a more potent product with fewer contaminants would allow for a lower dose in patients, potentially reducing both the risk of adverse effects and overall treatment costs.

## Concluding Remarks

In this study, we demonstrate that the efficacy of current bothropic antivenom for treating snakebite envenomation can be significantly improved using affinity chromatography purification. While alternative technologies are under development to create next-generation antivenoms, existing antivenom therapies will remain essential for years to come. Our findings quantitatively show that a relatively simple affinity chromatography step can yield an antivenom enriched in neutralizing F(ab’)_2_ and can significantly improve the efficacy of these current treatments.

## Data Availability

Mass spectrometry data were deposited to the ProteomeXchange Consortium via the MassIVE repository with the data set identifier MSV000096324.

## Supporting Information Available

Quantitative proteomic analysis of the bothropic antivenom (BAv) and of the improved bothropic antivenom (iBAv) (Table S1) (XLSX)
